# Supplementary material for: Identification of photoperiod-induced specific miRNAs in the adrenal glands of Sunite sheep (Ovis aries)
Source: Front Vet Sci. 2022 Jul 22;9:888207. doi: 10.3389/fvets.2022.888207 (PMC9354845; doi:10.3389/fvets.2022.888207)
Supplement: Supplementary file 1 [file Data_Sheet_1.ZIP › Supplementary file/miRDeep2 file.zip!/miRDeep2 file/Novel_Structure/SPLP42A2/Novel_665.pdf]

Provisional ID	:	NC_019458.2_1692
Score total	:	1.4
Score for star read(s)	:	-1.3
Score for read counts	:	0
Score for mfe	:	1.1
Score for randfold	:	1.6
Score for cons. seed	:	
Total read count	:	3476
Mature read count	:	3476
Loop read count	:	0
Star read count	:	0

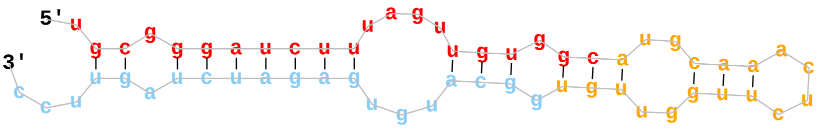

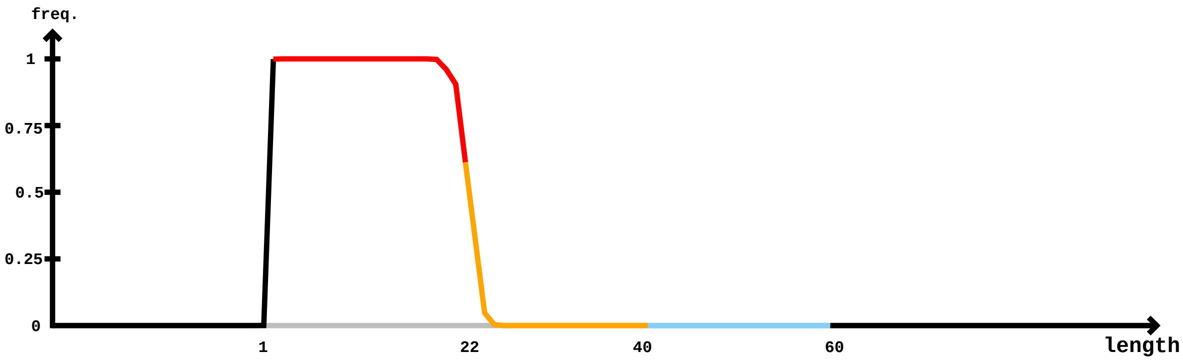

Mature **Star**

```
5'- ucgucagucuuuguuuuggguugcgggaucuuuaguuguggcaugcaaacucuugguuguggcaugugagaucuaguuccccaacaagggauggaugcuuacuccuuggaaga  -3'    exp
    .((((.((((((...(((((..((.(((((((....(((.(((..(((....)))..))).)))...))))))).))..)))))..)))))).))))((..(.....)..)).  reads      mm        sample
    ....................uugcgggaucuuuaguuguggca......................................................................  1          0         seq
    .....................ugcgggaucuuuaguuA...........................................................................  5          1         seq
    .....................ugcgggaucuuuaguug...........................................................................  2          0         seq
    .....................ugcgggaucuuuaguugC..........................................................................  21         1         seq
    .....................ugcgggaucuuuaguugu..........................................................................  99         0         seq
    .....................ugcgAgaucuuuaguugu..........................................................................  1          1         seq
    .....................ugcAggaucuuuaguugu..........................................................................  3          1         seq
    .....................ugcgggaucuuuaguugA..........................................................................  6          1         seq
    .....................ugcgggaucuuuaguugug.........................................................................  150        0         seq
    .....................ugUgggaucuuuaguugug.........................................................................  1          1         seq
    .....................ugcgggaucuuuaguuguA.........................................................................  40         1         seq
    .....................ugcgAgaucuuuaguugug.........................................................................  1          1         seq
    .....................ugcgggaucuuuaguugCg.........................................................................  2          1         seq
    .....................ugcgggaucuuuaguugugU........................................................................  54         1         seq
    .....................ugcgggaucuuuaguugugA........................................................................  224        1         seq
    .....................ugcgggaucuuuagCugugg........................................................................  1          1         seq
    .....................ugcgggaucuuuaguugCgg........................................................................  5          1         seq
    .....................ugcgggaucuuuaguugugg........................................................................  718        0         seq
    .....................ugcgggaucuuuaguuguAg........................................................................  4          1         seq
    .....................ugGgggaucuuuaguugugg........................................................................  1          1         seq
    .....................ugcgggaucuuuUguugugg........................................................................  1          1         seq
    .....................ugUgggaucuuuaguugugg........................................................................  4          1         seq
    .....................ugcggAaucuuuaguugugg........................................................................  1          1         seq
    .....................ugcAggaucuuuaguugugg........................................................................  4          1         seq
    .....................ugUgggaucuuuaguuguggc.......................................................................  6          1         seq
    .....................ugcgggaucuuuaguugugAc.......................................................................  7          1         seq
    .....................ugcgggaucuuuaguuguAgc.......................................................................  3          1         seq
    .....................ugcgggaucuuuaguugAggc.......................................................................  1          1         seq
    .....................ugcgggaucuuuaguuguggU.......................................................................  519        1         seq
    .....................ugcgggaucuuuaguugCggc.......................................................................  4          1         seq
    .....................ugcgggaucuuuUguuguggc.......................................................................  1          1         seq
    .....................ugGgggaucuuuaguuguggc.......................................................................  1          1         seq
    .....................ugcgggaucuuuaguuguggc.......................................................................  1355       0         seq
    .....................ugcgggaucuuuagCuguggc.......................................................................  1          1         seq
```

```
                    Mature                                  Star
ucgucagucuuuguuuuggguugcgggaucuuuaguuguggcaugcaaacucuugguuguggcaugugagaucuaguuccccaacaagggauggaugcuuacuccuuggaaga
.....................ugcgggGucuuuaguuguggca......................................................................    6     1     seq
.....................ugcgggaucuuuaguuguAgca......................................................................    2     1     seq
.....................uAcgggaucuuuaguuguggca......................................................................    10    1     seq
.....................ugcgAgaucuuuaguuguggca......................................................................    5     1     seq
.....................ugAgggaucuuuaguuguggca......................................................................    1     1     seq
.....................ugcAggaucuuuaguuguggca......................................................................    18    1     seq
.....................ugUgggaucuuuaguuguggca......................................................................    4     1     seq
.....................ugGgggaucuuuaguuguggca......................................................................    3     1     seq
.....................ugcgggaucuuuaguuguggUa......................................................................    11    1     seq
.....................ugcgggaucuuuaguugCggcau.....................................................................    1     1     seq
.....................ugcgggaucuuuaguuguggcaC.....................................................................    12    1     seq
.....................ugcgggaucuuuaguuguggcau.....................................................................    138   0     seq
.....................uAcgggaucuuuaguuguggcau.....................................................................    2     1     seq
.....................ugcgggaucuuuaguuguggcaug....................................................................    1     0     seq
.....................ugcgggaucuuuaguuguggcauU....................................................................    9     1     seq
.....................ugcgggaucuuuaguuguggcauC....................................................................    1     1     seq
.....................ugcgggaucuuuaguuguggcauA....................................................................    2     1     seq
......................gcgggaucuuuaguuguggU.......................................................................    1     1     seq
......................gcgggaucuuuaguuguggca......................................................................    2     0     seq
```
